# Knowledge of and Testing Rate for Hepatitis C Infection among the General Public of Saudi Arabia: A Cross-Sectional Study

**DOI:** 10.3390/ijerph20032080

**Published:** 2023-01-23

**Authors:** Mohammad S. Alzahrani, Abdullah Ayn Aldeen, Rayan S. Almalki, Mohammed B. Algethami, Nawaf F. Altowairqi, Ammar Alzahrani, Ahmed S. Almalki, Rami M. Alzhrani, Majed A. Algarni

**Affiliations:** 1 Department of Clinical Pharmacy, College of Pharmacy, Taif University, Taif 21944, Saudi Arabia; 2 College of Pharmacy, Taif University, Taif 21944, Saudi Arabia; 3 Faculty of Medicine, King Abdulaziz University, Jeddah 22252, Saudi Arabia; 4 Department of Pharmaceutics and Industrial Pharmacy, College of Pharmacy, Taif University, Taif 21944, Saudi Arabia

**Keywords:** knowledge, public health, chronic disease, hepatitis C, Saudi Arabia

## Abstract

Introduction: The Ministry of Health in Saudi Arabia has announced a plan to eradicate hepatitis C virus (HCV) infection. This study sought to evaluate the knowledge levels and testing rate among the general population of Saudi Arabia. Methods: A cross-sectional study was conducted using data collected from an online, self-administered survey. Multivariable analysis was conducted using multiple binary logistic regression models to identify factors associated with low knowledge levels as well as predictors of HCV testing. Results: A total of 689 participants completed the survey. While most participants (88%) have heard of HCV infection, less than half (47.3%) understood that HCV is curable with medications. More than half of the participants (53.7%) have low knowledge about HCV infection. Testing for HCV was reported by 123 respondents (17.8%), and the odds of testing for HCV were significantly lower among residents of the Makkah region (OR = 0.59 [95% CI: 0.36–0.97]) and those with low knowledge level (OR = 0.47 [95% CI: 0.29–0.74]). HCV diagnosis was reported by nine respondents (1.3%), of whom only four reported receiving treatment (44%). Conclusions: Our study indicates inadequate knowledge levels and relatively low testing rate. These findings underscore the need for national awareness campaigns and more effective strategies for HCV screening.

## 1. Introduction

Hepatitis C virus (HCV) infection is a major public health issue. An estimated 58 million individuals worldwide are chronically infected with HCV, with 1.5 million new infections occurring each year [1]. According to the World Health Organization, over 290,000 individuals died from hepatitis C in 2019 [1]. It is estimated that around 3% of the world population has a HCV infection, with higher prevalence in some groups such as injected drug users (IDUs) and HIV positive populations [2]. HCV patients remain asymptomatic for years [3], with the possibility of developing cirrhosis or hepatocarcinoma [4].

In Saudi Arabia, HCV varies by province, with the western and southern provinces having the highest rates [5]. HCV prevalence is reportedly declining among blood donors due to increased awareness and improved socioeconomic position [5]. The seroprevalence rate of HCV was estimated to be 1.1% based on data from Saudi blood donors [6]. HCV is still a significant public health issue in Saudi Arabia, especially among intravenous drug users and hemodialysis patients [7].

The introduction of new, safe, and effective direct-acting antivirals (DAAs) in 2011 represented a major development in the treatment of HCV with a response exceeding 90% [8,9]. DAAs have changed the treatment of HCV, almost completely removing old, poorly tolerated treatments (e.g., interferon and ribavirin), offering a cure for most patients [10].

The knowledge levels of HCV infection have previously been evaluated in the literature in various countries. Habtemu et al. conducted a cross-sectional study in Ethiopia that reported that most participants (60.9%) had a good knowledge level of HCV infection [11]. Another study that was conducted among HCV patients in Egypt found that about half of the patients (49.7%) had a good knowledge about HCV infection [12]. Prior studies have found that knowledge levels can vary with certain facts about HCV infection. A study that was conducted among African American HCV patients in the United States reported that about 82% of the study participants answered correctly to knowledge items related to transmission through needle sharing, while only 12.4% answered correctly to items related to HCV vaccination [13]. Other studies sought to compare the knowledge of HCV infection compared with other similar infections. A previous study showed that knowledge among health workers in Malawi on HCV was less compared with HIV/AIDS [14].

Few studies have been conducted among the general population of Saudi Arabia with regard to their knowledge of HCV infection. A prior study focused on Saudi dental patients reported that more than 60% of the participants were aware of both hepatitis B virus and HCV [15]. Another study that was conducted in 2014–2015 among people visiting primary health care centers in Jeddah city, Saudi Arabia, found that the level of knowledge of HCV was low, particularly knowledge related to HCV prevention and treatment [16]. The Ministry of Health (MOH) in Saudi Arabia launched a national campaign for HCV detection and treatment in 2018 [17]. One goal of this program is to train doctors and directors and provide lab and radiology tests as well as other logistic services that are needed to implement the campaign [17]. Raising the public’s awareness of HCV is also one of the main goals [17]. With the evolving treatment landscape in HCV infection, a timely assessment of HCV knowledge and testing is needed. Hence, the objectives of this study were to evaluate the awareness and knowledge of HCV infection among the general population of Saudi Arabia, estimate the testing rate for HCV infection, and evaluate HCV treatment uptake among participants who reported a HCV diagnosis.

## 2. Materials and Methods

### 2.1. Study Design and Setting

This was a cross-sectional study design using data collected from an online, self-administered survey from 26 January 2022 through to 26 February 2022. The eligible population included people who were 18 years or older and who resided in Saudi Arabia. In this study, participants were recruited through a non-probability snowball sampling technique, where a link to the web-based survey was promoted first by the authors on social media and then the participants were asked to share it with others. The survey was administered using Google Forms, and it was designed to ensure that each participant could only respond to the survey once. Written informed consent was collected from each participant before starting the survey. No personal information was collected in the survey, and the confidentiality of the responses was maintained. The sample size was determined using the formula N = Z^2^ P (1 − P)/d^2^, where Z is the level of confidence, P is the estimated prevalence, and d is the margin of error [18]. With prevalence set at 1.1% ± 0.6%, as suggested in a previous study [6], the minimum sample size needed to obtain 80% power was 496.

### 2.2. Data Collection

The survey tool that was used in this study comprised of three sections. The first section included items about the sociodemographic characteristics. We collected data about age, gender, marital status, nationality, education level, income, geographical region, and whether the respondent was working or studying in the medical field. The second section included 11 items regarding the knowledge of HCV infection. One item asked whether the respondents had ever heard about HCV infection. The other 10 items covered a range of facts about the transmission of HCV infection, its long-term health complications, and the availability of a cure or vaccine for HCV infection. These items were adapted from similar studies [12,13]. Each item has three possible answers (“True”, “False”, and “Don’t know”). Correct responses were given points and then added up together to calculate the total knowledge score for each respondent, with a total score ranging from 0 to 10. Using the median score as a cut-off point, knowledge levels were recoded into two categories (low vs. high knowledge). Those with a total score equal or less than the median were considered as having a low knowledge level. The third section of the survey tool included items regarding whether the respondents had been tested before for HCV infection, the reasons for doing the test, whether they or someone they knew had been diagnosed with HCV infection, and whether those who had been diagnosed with HCV infection had received any treatment. In total, the questionnaire had 26 items, and it took about 20 min or less to complete. The survey tool was assessed for face validity by two faculty members with knowledge on the subject.

### 2.3. Statistical Analysis

Univariable, bivariable, and multivariable analyses were performed. The mean and median were calculated for the numerical variables. Frequency and percentages were calculated for the categorical variables. Bivariable analysis was conducted using chi-square or Fisher’s exact test where appropriate to assess the association with the outcomes, HCV knowledge, and HCV testing. Simple and multiple logistic regressions were performed to identify factors associated with low knowledge levels and predictors of HCV testing. Odds ratio (OR), along with their 95% confidence interval (CI), were calculated to determine the significance and strength of association with the outcome. *p*-Values of less than 0.05 were considered statistically significant. All statistical analyses were performed using SAS^®^ University Edition.

## 3. Results

### 3.1. Characteristics of Respondents

A total of 689 participants completed the online survey. Table 1 shows the distribution of respondents by demographic characteristics. The age of the respondents ranged from 18 to 66 years, with a mean age of 34.1 ± 11.9 years. The majority of participants were male (62%), bachelor’s degree holders (65%), of Saudi nationality (96%), and living in the Makkah region (81%). Less than one-third of participants (30.5%) reported that they knew someone infected with HCV. Only 1.31% (n = 9) of the study respondents reported HCV infection diagnosis, of whom less than half (n = 4) reported receiving treatment.

### 3.2. Knowledge of HCV Infection among Respondents

The majority of respondents (88%) had heard of HCV infection. Table 2 shows the respondents’ knowledge about HCV infection. Only about 40% of respondents believed that a person infected with HCV could live without symptoms or health problems for years. In contrast, nearly two-thirds believed that HCV infection could cause other diseases such as liver cirrhosis and liver cancer. Only about 20% knew that there was no vaccine for HCV infection, and less than half knew that HCV infection could be completely cured with medications.

Knowledge levels by characteristics of the study respondents are shown in Table 3. Overall, more than half of the study respondents (54%) had a low knowledge level (score ≤ median). About 59% of female respondents had a low knowledge level compared to 50% of the male respondents (*p* = 0.021). More than two-thirds of the respondents with high school or less had a low knowledge level. By income level, low knowledge was significantly more prevalent among those in the low-income category (*p* = 0.014). Low knowledge was also more prevalent among respondents who were not working or studying in the medical field and among those who did not report knowing someone infected with HCV infection (*p* < 0.001).

Table 4 shows the results of the simple and multiple logistic regression analyses of the HCV knowledge levels. Compared to the male respondents, female respondents were more likely to have a low knowledge level (unadjusted OR = 1.44 [95% CI: 1.06–1.96], *p* = 0.021). However, gender was not significantly associated with HCV knowledge after adjusting for the other covariates. The adjusted model showed that the odds of having low HCV knowledge were greater among those with high school or less (vs. those with higher education) and among respondents with low income (vs. high income). Not testing for HCV was significantly associated with greater odds of having low HCV knowledge (adjusted OR = 2.19 [95% CI: 1.39–3.44], *p* = 0.001).

### 3.3. Testing for HCV among Respondents

**Figure 1:**
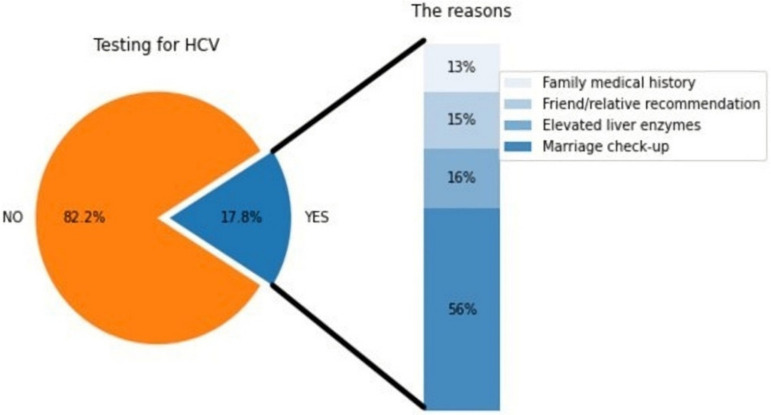
Self-reported HCV testing rate and the reasons for being tested.

**Figure 2:**
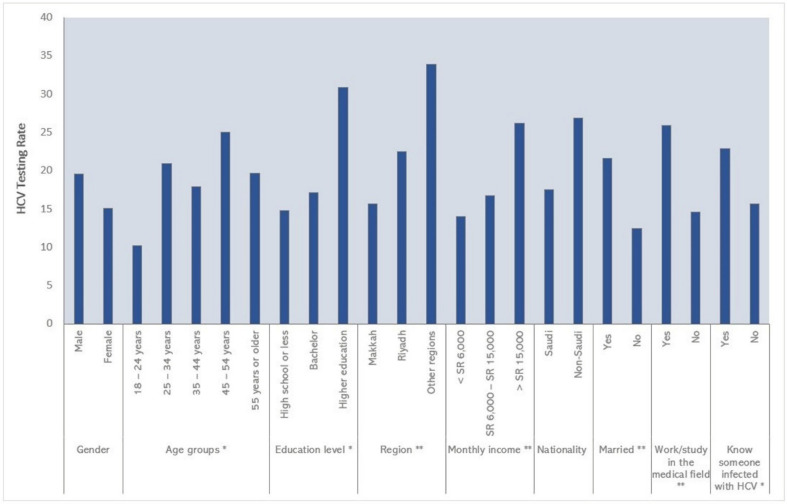
Self-reported HCV testing rates by the characteristics of the study respondents. * indicates significant differences with *p*-value < 0.05, ** indicates significant differences with *p*-values < 0.01.

Testing for HCV was reported by only 17.8% (n = 123). The most frequent reason for HCV testing was marriage health check-up, followed by medical reasons such as elevated liver enzymes and family medical history of liver disease (Figure 1). HCV testing rates varied significantly by age groups (*p* = 0.014), with the highest rate reported by those who were 45–54 years old (Figure 2). By geographical region, the Makkah region had the lowest HCV testing rate (15%) compared to Riyadh (22%) and other regions (33%) (*p* = 0.001). Additionally, the HCV testing rates differed significantly by education level (*p* = 0.01), marital status (*p* = 0.002), and monthly income (*p* = 0.004). Respondents who were working or studying in the medical field and those who knew someone infected with HCV had significantly higher testing rates.

The predictors of testing for HCV are shown in Table 5. The multiple logistic regression analysis showed that residents of the Makkah region were less likely to get tested for HCV compared to their counterparts from Riyadh and other regions (adjusted OR = 0.59 [95% CI: 0.36–0.97], *p* = 0.038). Additionally, having low HCV knowledge was significantly associated with lower odds of getting tested for HCV. Being married was a significant predictor of getting tested for HCV (adjusted OR = 2.45 [95% CI: 1.20–4.96], *p* = 0.013). Working or studying in the medical field was another significant predictor of testing for HCV (adjusted OR = 2.19 [95% CI: 1.31–3.69], *p* = 0.003).

## 4. Discussion

The recent availability of a cure for HCV infection makes eliminating this disease more possible than ever. There is a global goal to eradicate HCV infection, and the MOH in Saudi Arabia has announced a national plan to achieve this goal [19]. One of the major obstacles to achieve this goal is public awareness. This study evaluated the levels of awareness and knowledge of HCV infection with the aim to identify populations with low knowledge levels. Hence, this study should guide decision-makers in Saudi Arabia in their future efforts to raise awareness of this infection. In addition to raising the awareness of HCV infection, it is necessary to encourage more people to get tested. The test for HCV infection is a reliable and cheap test. In this study, we estimated the HCV testing rate and described the reasons for testing. Furthermore, this study is among the first to estimate treatment uptake among those diagnosed with HCV infection in Saudi Arabia.

In this study, we found that the self-reported HCV infection was 1.3%. This finding agreed with a previous study that reported a prevalence rate of 1.1% [6]. The overall knowledge of HCV infection in our sample was low, with 54% of respondents having low knowledge levels. A previous study that was conducted in Egypt and also used the median as a cut-off point found 50.3% had low knowledge [12]. Most of the previous studies that were conducted in Saudi Arabia were limited to a certain population or geographical region. A prior study that was conducted in Saudi Arabia and published in 2021 concluded that 42% of participants showed low knowledge about viral hepatitis in general [20]. Another study that was conducted in the Riyadh region among dental students found that 37% of the students did not have adequate knowledge about HCV infection [21]. While we found that the majority had heard of HCV infection, less than half (47.5%) believed that HCV infection is curable with medications, and only 20% of participants knew that there was no vaccine for HCV infection. This poor knowledge of HCV infection among the Saudi population agreed with the results of another study that was conducted in 2014–2015 in Jeddah city, Saudi Arabia, where the authors stated that “The level of knowledge of the natural history, risk behavior, and prevention and treatment of HCV was poor among the participants” [16]. Knowing that the most prevalent HCV genotype in Saudi Arabia is genotype 4 [6], which is highly pathogenic, some poor knowledge of the participants may represent a risk factor for the spread of HCV infection. For example, more than 60% did not believe that HCV could be transmitted via sexual relationships. We also found that being a female was associated with having lower knowledge about HCV infection despite the fact that the general female population in Saudi Arabia have similar education levels as their male counterparts [22]. After adjusting for other factors, however, gender was no longer a significant predictor of knowledge about HCV infection.

In our study, we found that the HCV testing rate was approximately 18%. We found that most of the testing rate (56%) was because of the mandatory premarital screening program or other special cases such as elevated liver enzymes. As clarified earlier, those who are married are more than two times more likely to have been tested for HCV. This finding goes hand in hand with another published article about HCV in Saudi Arabia in 2018, which stated that 15% of new cases would likely be diagnosed via premarital screening [23]. The study also suggested that it is a more cost-effective way than screening the whole population [23]. Premarital hepatitis screening started in 2008 as a mandatory screening along with other infections [24]. As expected, workers in the medical field and those with higher HCV knowledge or higher education were more likely to get tested for HCV. Those with higher monthly income were more likely to be knowledgeable and to get tested, and the explanation for that could be that they have more access to information sources, are more concerned with their well-being, and they can afford the test. We also found that knowledge about HCV infection is a significant predictor of HCV testing. Those with low knowledge were less likely to get tested for HCV. This finding highlights the importance of the ongoing national awareness campaigns.

Our study showed that the treatment uptake for HCV-infected patients in Saudi Arabia is suboptimal, with a treatment rate of about 44%. This finding could mean that Saudi Arabia may miss the goal of eradicating hepatitis by 2030, which was set by the WHO [25]. Future studies are needed to further evaluate the treatment uptake of HCV infection in Saudi Arabia.

There are several limitations that need to be acknowledged. First, most of the participants in this study were from the Makkah region, and male participants were the majority. Using a non-probability sampling technique may have led to selection bias. Thus, the generalizability of the study findings is limited. Second, there is a potential risk of recall bias as some questions require recalling some events in the past. Third, because of the small number of respondents who reported HCV diagnosis, we were unable to evaluate the factors affecting treatment uptake.

## 5. Conclusions

Our findings suggest that knowledge about HCV infection among the general public of Saudi Arabia is inadequate. This study also revealed that the testing rate was relatively low and knowledge about HCV infection was an independent predictor of HCV testing. For a disease that is now curable, we found that treatment uptake was suboptimal among those who reported HCV diagnosis. In order to achieve the goal of HCV eradication, more national awareness campaigns and more effective strategies for national HCV screening and treatment are needed. Future studies may need to further assess the reasons behind the regional differences in knowledge as it is the cornerstone for HCV eradication. Additionally, future studies may need to assess the factors associated with treatment uptake in the Saudi population.

## Figures and Tables

**Table 1:** Characteristics of the study participants (N = 689).

Characteristics	n (%)
Gender	
Male	424 (61.54)
Female	265 (38.46)
Age, mean ± SD	34.1 ± 11.9
Age groups	
18–24 years	186 (27)
25–34 years	163 (23.66)
35–44 years	156 (22.64)
45–54 years	108 (15.67)
55 years or older	76 (11.03)
Education level	
High school or less	176 (25.54)
Bachelor	445 (64.59)
Higher education	68 (9.87)
Region	
Makkah	562 (81.57)
Riyadh	71 (10.3)
Other regions	56 (8.13)
Marital status	
Single	271 (39.33)
Married	402 (58.35)
Divorced	14 (2.03)
Widowed	2 (0.29)
Monthly income	
<SR 6000	285 (41.36)
SR 6000–SR 15,000	240 (34.83)
>SR 15,000	164 (23.80)
Nationality (Saudi)	663 (96.23)
Work/study in the medical field (Yes)	197 (28.60)
Know someone infected with HCV (yes)	210 (30.50)
Ever heard of HCV infection (Yes)	604 (88)
Self-reported HCV infection diagnosis (Yes)	9 (1.31)

**Table 2:** Knowledge statements about HCV infection.

Item No.	Item	Responses		
		Yes	No	Don’t Know
1	A person with hepatitis C can live for	277 *	179	233
	years without symptoms or health problems	(40.2%)	(25.9%)	(33.8%)
2	Hepatitis C can cause other diseases	447 *	36	206
	such as liver cirrhosis and liver cancer	(64.8%)	(5.2%)	(29.9%)
3	Hepatitis C can be transmitted through	482 *	56	151
	blood transfusion from an infected person	(69.9%)	(8.1%)	(21.9%)
4	This disease can be passed on via sex	262 *	209	218
	with an infected person	(38%)	(30.3%)	(31.6%)
5	This disease can be spread through	361 *	115	213
	cupping or piercing	(52.4%)	(16.7%)	(30.9%)
6	This disease can be transmitted by using personal hygiene products from an infected person, such as toothbrushes and razors	394 * (57.1%)	132 (19.2%)	163 (23.7%)
7	It is possible to transmit the disease through a casual contact with an infected person such as shaking hands	32 (4.6%)	514 * (74.6%)	143 (20.7%)
8	It is possible to transmit the disease through an infected mother to her child during childbirth	240 * (34.8%)	163 (23.6%)	286 (41.5%)
9	There is a vaccine for hepatitis C	333	135 *	221
		(48.3%)	(19.6%)	(32.1%)
10	Hepatitis C can be completely cured	326 *	103	260
	with medications	(47.3%)	(14.9%)	(37.7%)

* Indicates correct responses.

**Table 3:** HCV knowledge levels by the characteristics of the study respondents.

Characteristics	HCV Knowledge Levels		
	Low Knowledge (Score ≤ Median), n (%)	High Knowledge (Score > Median), n (%)	*p*
Overall	370 (53.7)	319 (46.3)	-
Gender			0.021
Male	213 (50.2)	211 (49.8)	
Female	157 (59.3)	108 (40.7)	
Age groups			0.886
18–24 years	95 (51.1)	91 (48.9)	
25–34 years	86 (52.8)	77 (47.2)	
35–44 years	88 (56.4)	68 (43.5)	
45–54 years	59 (54.6)	49 (45.4)	
55 years or older	42 (55.3)	34 (44.7)	
Education level			<0.001
High school or less	118 (67.1)	58 (32.9)	
Bachelor	223 (50.1)	222 (49.9)	
Higher education	29 (42.7)	39 (57.3)	
Region			0.148
Makkah	311 (55.3)	251 (44.7)	
Riyadh	35 (49.3)	36 (50.7)	
Other regions	24 (42.9)	32 (57.1)	
Marital status			0.019
Married	231 (57.5)	171 (42.5)	
Single\Divorced\Widowed	139 (48.4)	148 (51.6)	
Monthly income			0.014
<SR 6000	169 (59.3)	116 (40.7)	
SR 6000–SR 15,000	127 (52.9)	113 (47.1)	
>SR 15,000	74 (45.1)	90 (54.9)	
Nationality			0.987
Saudi	356 (53.7)	307 (46.3)	
Non-Saudi	14 (53.9)	12 (46.1)	
Work/study in the medical field			<0.001
Yes	56 (28.4)	141 (71.6)	
No	314 (63.8)	178 (36.2)	
Know someone infected with HCV			<0.001
Yes	85 (40.5)	125 (59.5)	
No	285 (59.5)	194 (40.5)	
Tested for HCV			<0.001
Yes	42 (34.2)	81 (65.8)	
No	328 (57.9)	238 (42.1)	

**Table 4:** Factors associated with low knowledge level.

Characteristics	Unadjusted OR [95% CI]	*p*	Adjusted OR [95% CI]	*p*
Gender				
Male	Ref.		Ref.	
Female	1.44 [1.06–1.96]	0.021	1.01 [0.69–1.46]	0.942
Age groups				
18–24 years	Ref.		Ref.	
25–34 years	1.07 [0.70–1.63]	0.726	1.19 [0.69–2.05]	0.239
35–44 years	1.24 [0.81–1.90]	0.518	0.79 [0.38–1.66]	0.331
45–54 years	1.15 [0.72–1.85]	0.887	0.81 [0.34–1.89]	0.704
55 years or older	1.18 [0.69–2.02]	0.801	0.93 [0.38–2.34]	0.49
Education level				
Higher education	Ref.		Ref.	
Bachelor	1.35 [0.81–2.26]	0.246	1.21 [0.67–2.18]	0.586
High school or less	2.74 [1.54–4.85]	<0.001	1.82 [0.95–3.46]	0.03
Region				
Riyadh and other regions	Ref.		Ref.	
Makkah	1.43 [0.97–2.10]	0.071	1.13 [0.72–1.75]	0.6
Marital status				
Single\Divorced\Widowed	Ref.		Ref.	
Married	1.44 [1.06–1.95]	0.019	1.66 [0.94–2.92]	0.08
Monthly income				
>SR 15,000	Ref.		Ref.	
SR 6000–SR 15,000	1.37 [0.92–2.04]	0.870	1.36 [0.85–2.17]	0.598
<SR 6000	1.77 [1.20–2.61]	0.008	2.27 [1.29–4.01]	0.005
Nationality				
Saudi	Ref.		Ref.	
Non-Saudi	1.01 [0.46–2.21]	0.987	0.94 [0.37–2.34]	0.893
Work/study in the medical field				
Yes	Ref.		Ref.	
No	4.44 [3.09–6.36]	<0.001	4.28 [2.80–6.53]	<0.001
Know someone infected with HCV				
Yes	Ref.		Ref.	
No	2.16 [1.55–3.01]	<0.001	2.26 [1.56–3.26]	<0.001
Tested for HCV				
Yes	Ref.		Ref.	
No	2.66 [1.77–3.99]	<0.001	2.19 [1.39–3.44]	0.001

**Table 5:** Predictors of testing for HCV.

Characteristics	Unadjusted OR [95% CI]	*p*	Adjusted OR [95% CI]	*p*
Gender				
Male	Ref.		Ref.	
Female	0.73 [0.48–1.10]	0.136	1.05 [0.65–1.70]	0.829
Age groups				
18–24 years	Ref.		Ref.	
25–34 years	2.32 [1.26–4.24]	0.344	1.82 [0.89–3.72]	0.244
35–44 years	1.92 [1.03–3.59]	0.941	1.24 [0.47–3.22]	0.553
45–54 years	2.93 [1.54–5.57]	0.043	1.75 [0.64–4.75]	0.405
55 years or older	2.16 [1.03–4.52]	0.676	1.46 [0.49–4.34]	0.93
Education level				
Higher education	Ref.		Ref.	
Bachelor	0.46 [0.26–0.81]	0.153	0.67 [0.35–1.28]	0.757
High school or less	0.39 [0.20–0.75]	0.029	0.53 [0.25–1.09]	0.117
Region				
Riyadh and other regions	Ref.		Ref.	
Makkah	0.49 [0.31–0.77]	0.002	0.59 [0.36–0.97]	0.038
Marital status				
Single\Divorced\Widowed	Ref.		Ref.	
Married	1.93 [1.26–2.93]	0.002	2.45 [1.20–4.96]	0.013
Monthly income				
>SR 15,000	Ref.		Ref.	
SR 6000–SR 15,000	0.56 [0.35–0.92]	0.381	0.74 [0.42–1.28]	0.216
<SR 6000	0.45 [0.28–0.74]	0.02	0.96 [0.49–1.90]	0.698
Nationality				
Saudi	Ref.		Ref.	
Non-Saudi	1.74 [0.71–4.22]	0.223	1.59 [0.58–4.38]	0.366
Work/study in the medical field				
No	Ref.		Ref.	
Yes	2.04 [1.36–3.05]	0.001	2.19 [1.31–3.69]	0.003
Know someone infected with HCV				
No	Ref.		Ref.	
Yes	1.59 [1.06–2.39]	0.024	1.09 [0.70–1.71]	0.682
HCV knowledge level				
High	Ref.		Ref.	
Low	0.38 [0.25–0.56]	<0.001	0.47 [0.29–0.74]	0.001

## Data Availability

Data are available upon reasonable request to the corresponding author.
